# The effects of sleeve gastrectomy on hormonal regulation of glucose metabolism in Goto–Kakizaki rats

**DOI:** 10.1007/s10353-014-0270-z

**Published:** 2014-07-10

**Authors:** Z. Zhu, X. Yang, K. Wang, Z. Wang, Y. Zhao, M. Yu

**Affiliations:** 1 Department of Plastic Surgery, Renmin Hospital of Wuhan University, 238#, Jiefang Road, 430060 Wuhan, Hubei People’s Republic of China; 2 Department of Otorhinolaryngology, Renmin Hospital of Wuhan University, 238#, Jiefang Road, 430060 Wuhan, Hubei People’s Republic of China

**Keywords:** Sleeve gastrectomy, Glucose metabolism, Type 2 diabetes mellitus, Insulin resistance, Gastrointestinal hormones

## Abstract

**Background:**

The antidiabetic effect of sleeve gastrectomy (SG) has been interpreted as a conceivable result of surgically induced weight loss in the obese type 2 diabetes mellitus (T2DM) subjects. However, the blood glucose control often occurs within days, before significant weight loss has been reached. This work aims to investigate the major mechanism and persistence regarding how SG improves glucose metabolism in nonobese T2DM rats.

**Methods:**

These Goto Kakizaki rats (*n* = 21) were randomly assigned into three groups: SG, sham SG, and pair-fed (PF) group, whose weight, food intake, oral glucose tolerance test, insulin tolerance test, plasma insulin, homeostasis model assessment for insulin resistance (HOMA-IR), ghrelin, and glucagon-like peptide-1 (GLP-1) were measured.

**Results:**

According to the experiment, from the 2nd week until the 24th week, the fasting blood glucose of the rats in the SG group had significantly decreased with the improved glucose tolerance. At the 2nd week postoperation, the area under the blood glucose concentration curve (AUC) received a distinct reduction of 28.1 % (*P* < 0.0001). The ghrelin secretion of the SG group was significantly decreased (*P* < 0.005). The GLP-1 had increased (*P* < 0.0001), while the HOMA-IR values decreased (*P* < 0.05) throughout the experimental period. These effects were not seen in the sham-SG and PF groups despite similar changes of weight loss or food intake.

**Conclusions:**

The above results suggest that SG can conduct a direct control on T2DM instead of secondarily to weight loss or food intake around the whole experimental period. The changes of the gastrointestinal hormones may be the major mechanism of the antidiabetic effect.

## Introduction

At present, diabetes mellitus (DM) is considered as a major risk factor for morbidity and mortality worldwide [1], which affects more than 371 million people worldwide [2] and collectively accounts for an estimated 12.9 million deaths globally in 2010 [3, 4]. In the most populous countries, the morbidity and the mortality of DM have been increasing rapidly, especially China contributed to this pandemic [2, 5, 6]. More than 90 % of the DM patients suffer from type 2 DM (T2DM) with the global burden of T2DM clearly increasing [7]. However, the etiology and best treatment still remain elusive.

Currently, bariatric surgery results in better glucose control than did medical therapy for T2DM [8]. The resolution of T2DM has been regarded as an outcome of surgical treatment of obesity [9, 10]. Approximately 20 years ago, biliopancreatic diversion (BPD) and Roux-en-Y gastric bypass (GBP) had a better effect on T2DM than other procedures, which can determine normal concentrations of plasma glucose, insulin, and glycosylated hemoglobin in 80–100 % of the morbid obese patients [11, 12]. Sleeve gastrectomy (SG) is proved to be a valid procedure with a lasting effect on weight loss [13]. Recently, researchers have found that SG is as effective as GBP in inducing remission of T2DM and metabolic syndrome (MS) within the severe obese subjects [14]. Overweight or obesity is the dominant risk factor for diabetes [15, 16], and weight loss or hypocaloric diet could reduce the plasma glucose and improve the insulin sensitivity of the obese individuals [17]. Therefore, the antidiabetic effect of surgery has been still interpreted as a conceivable result of the surgically induced weight loss and decreased caloric intake [9, 10].

Nevertheless, the glycemic control often occurs within days before significant weight loss has been reached [18, 19], which suggests that the control of the glycemic status may be a direct effect of the surgery rather than a secondary effect of the weight loss.

After either the BPD or GBP operation, the gastrointestinal (GI) hormones, such as ghrelin and glucagon-like peptide-1 (GLP-1) will be changed. These hormones might have been involved in regulating the beta cell function in both physiological [20] and pathophysiological status [21, 22]; thus, these changes in the enteroinsular axis maybe explained as the antidiabetic effect.

SG as described by Gagner et al. [23, 24], has recently emerged as a stand along bariatric procedure rather than just a gastric restrictive operation [25]. In addition, the date from the case series has shown that SG is associated with a high rate of resolution of the T2DM and the obesity-associated comorbidities such as hypertension, hyperlipidemia, and sleep apnea [26, 27], and is similar to the GBP in inducing remission of T2DM and the MS [7].

Despite a major risk factor for T2DM, obesity does not mean that all the patients with T2DM are obese. It is reasonable to assume that if the control of the diabetes was a direct effect of SG rather than a secondary result of the treatment of obesity; the similar outcome could also be observed in the nonobese individuals. To confirm this hypothesis, the present study is focused on the effect of SG on the Goto–Kakizaki (GK) rats, the most widely used animal model for nonobese T2DM [28].

## Materials and methods

### Animals

A total of 21 13-week-old male GK rats (National Rodent Laboratory Animal Resources, Shanghai, China), were individually housed under 22 °C and 60 % humidity in a 12-h light/dark cycle at Wuhan University. All rats had free access to tap water and were fed with standard rat chow diet. All procedures related to the animal experiments were approved by the Animal Care and Utilization Committee of Wuhan University.

### Experimental protocol

After being acclimated for 2 weeks, the weight, food intake, fast glucose, and oral glucose tolerance of the rats were measured. Then, the 15–16-week-old rats, randomly underwent one of the following procedures: SG (*n* = 9), sham-SG (sham-SG, *n* = 6), and pair-fed ((PF), *n* = 6). All groups were fed with the same type of diet. For SG, sham-SG, and PF animals, the food intake, weight changes, and fasting glucose were measured weekly for 24 weeks; the oral glucose tolerance test (OGTT) was measured at preoperative stage as well as in the 2nd, 4th, 10th, and 24th week after the surgery; the plasma insulin, ghrelin, and GLP-1 were measured at the preoperative stage as well as in the 2nd, 4th, 6th, 10th, 16th, and 24th week after the operation. The insulin tolerance test (ITT) was performed on rats in the 2nd and 6th week after the surgery.

### Intervention

The rats which had undergone both the SG and sham-SG were anesthetized with 1 % pentobarbital sodium without taking any food overnight. Before the operation, atropine (Shanghai Harvest Pharmaceutical, China, 125 mg/kg) and kanamycin sulfate (Amresco, American. 0.01 mg/kg) were intramuscularly injected in the hip, and 5 % glucose saline was subcutaneously injected during the surgery. After the operation, to prevent dehydration and infection, 10 ml of 5 % glucose in normal saline was injected subcutaneously in 7 days, and the antibiotic was intramuscularly injected in 3 days. Rats were kept 24 h without water and 3 days without food, the 1st day after the operation, all the animals were given a complex nutrient solution containing vitamin B complex and antibiotic; small amounts of food was allowed taking by the subject rats on the 3rd day after the operation. From the 7th day after the operation, the SG and sham-SG rats were fed with standard solid diet and tap water.

**Fig. 1:**
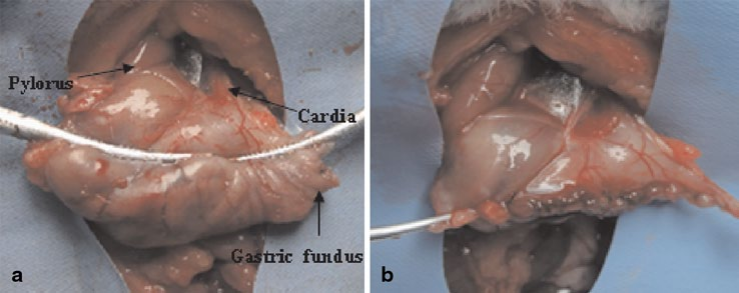
Sleeve gastrectomy: **a** Outlining of the area to be resected with microvascular clamp. **b** Removing approximately 70–80 % of stomach, including the whole gastric fundus and then following gastrorraphy with invaginating suture

For the rats of the SG, 70–80 % volume of the stomach was removed, which contained of the major part of the stomach and all the gastric fundus. The details of the procedure are illustrated in the Fig. 1a, and b.

For the sham-SG, the stomach was incised for 2–3 cm and immediately sutured. The operative time was prolonged to ensure an equivalent degree of anesthesiological stress on the rats that underwent SG.

For the PF, the PF group was given the same amount of food as the SG rats consumed.

### Methods

Weight and food intake of the rats were measured every week since the first postoperation month and on a fortnight basis afterward.

For the fasting blood glucose and fasting plasma insulin (FPI) concentration, the blood was collected from the orbital venous sinus of the conscious rats after a fasting period of 16–18 h. The samples were stored in ethylenediaminetetraacetic acid (EDTA) containing (1.5 µg/ml) tubes, and centrifuged at 3000 rpm at 4 °C for 15 min; then, these plasma samples were immediately separated and stored at − 80 °C until being analyzed. Fasting plasma glucose (FPG) was analyzed by the glucose oxidase method (BioSino Bio-technology and Science Inc, Beijing, China), and the FPI concentration was measured by enzyme-linked immunosorbent assay kits (Mercodia AB, Uppsala, Sweden).

Homeostasis model assessment of insulin resistance (HOMA-IR) is a less invasive, minor labor-intensive, and inexpensive method to measure compared with the euglycemic hyperinsulinemic clamp method [29]. Only the fasting glucose and fasting insulin concentration are needed in the method to evaluate the level of IR, which is used to calculate an index from the product of FPI (microunits per milliliter) and FPG (millimolar concentrations) divided by 22.5.

For the OGTT, after 16–18 h of fasting, blood glucose was measured in conscious rats before (baseline) and 30, 60, 120, 180 min after the administration of 3 g/kg glucose by oral gavage. The blood was obtained from the tail vein and analyzed with a glucometer (One Touch® Ultra, Lifescan, lnc.U.S.A. in the U.K.).

ITT was performed postoperative by measuring glucose levels before and 15, 30, 60, 120, and 180 min after injection of 0.5 UI/kg human insulin intraperitoneally in conscious fed rats.

For the plasma hormones measurements, the ghrelin level was measured after 16–18 h fasting, while the GLP-1 level was measured 30 min after the administration of 3 g/kg glucose by oral gavages. The blood samples from the orbital venous sinus of the conscious rats were collected in EDTA (1.5 µg/ml) tubes with the GI preservative (Aprotinin, 40 µg/ml). After centrifugation at 3000 rpm at 4 °C for 15 min, these plasma samples were immediately separated and stored at − 80 °C until being analyzed. Enzyme-linked immunosorbent assay kits were used for the measurement of the active ghrelin (ELISA; Linco Research, St. Charles, Missouri, United States) and active GLP-1 (Millipore, Billerica, MA).

### Statistical analysis

The Kolmogorov–Smirnov test was used to check the assumption of normal distribution in each group. There was no evidence for non-normality in any group. The data were expressed as mean ± standard deviation (SD). The areas under the curves of OGTT and ITT were calculated by the trapezoidal integration. Comparisons among the groups were made by using a one-way analysis of variance (ANOVA). A Student’s t-test was used wherever appropriate. Statistical significance levels were set at *P* < 0.05.

## Results

Before the treatments, body weight, fasting glucose, OGTT, the plasma insulin, ghrelin, and GLP-1 had no significant differences among the groups of GK rats.

After performing the SG on nine GK rats, sham-SG on six GK rats, all of the GK rats survived.

### Weight and food intake:

**Fig. 2:**
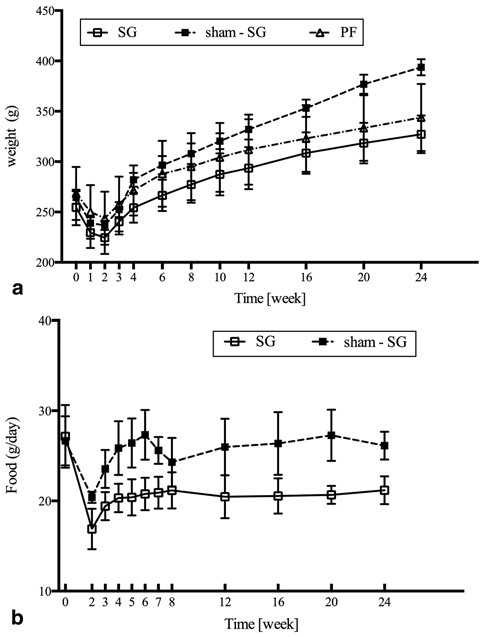
Weight changes and food intake: **a** Both the sleeve gastrectomy (SG) and pair-fed groups show more weight loss comparing with that of the sham-SG rats (*P* < 0.001). **b** SG group ate less food than sham-SG group (*P* < 0.001)

As shown in the Fig. 2a, the SG, sham–SG, and PF groups had similar weight loss at the 2nd week after the surgery. After four postoperative weeks, both the SG and PF groups had significantly more weight loss compared with sham-SG group (*P* < 0.001), which started regaining weight approximately from the 14th postoperative day. The mean weight loss of the SG and PF groups did not differ from one another at any period (*P* > 0.05). Due to surgical stress, the rats in the SG group ate less food than the rats in the sham-SG group (*P* < 0.001) (Fig. 2b). The SG and PF groups had the same average food intake throughout the study.

### Fasting glucose:

**Fig. 3:**
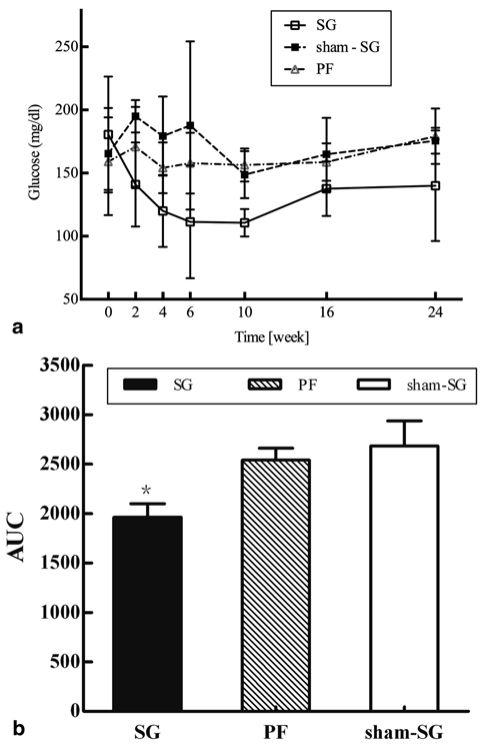
Fasting glucose: **a** Mean fasting glucose remained constantly lower in the sleeve gastrectomy (*SG*) group comparing with that of the *sham-SG* and pair-fed (*PF*) groups, the *PF* and *sham-SG* groups had no differences. **b** The *AUC* shows the area under the curve for fasting glucose over the 24-week period of postoperative observation among the *SG*, *sham–SG*, and *PF* groups (*P < 0.001)

SG group remarkably reduced the FPG levels. At the 10th week after the operation, the mean plasma glucose levels in the SG group were lower than before (110.6 ± 10.9 vs 148.8 ± 18.6 mg/dl, *P* = 0.002). However, the sham-SG and the PF groups did not significantly change blood glucose levels, and their glycemia remained consistently lower in the SG group with respect to the other two groups through the entire follow-up period (*P* < 0.001) (Fig. 3).

### Insulin:

The insulin concentrations of the three groups of the GK rats had no significant difference throughout the experiment (*P* > 0.05).

### Homeostasis model assessment of insulin resistance:

**Fig. 4:**
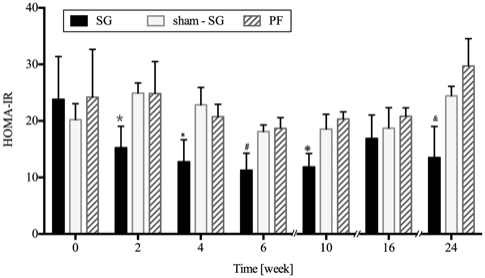
Mean ± standard deviation of insulin resistance (*HOMA-IR* homeostasis model assessment-insulin resistance) in the sleeve gastrectomy (*SG*), *sham-SG*, and pair-fed (*PF*) groups: *SG* group improved the insulin resistance during the 24-week period. *P < 0.034; †*P* < 0.026; #*P* < 0.008; * P* < 0.008; & *P* < 0.028

The study showed that the HOMA-IR of SG group was significantly lower than the sham-SG and the PF groups at 2nd, 4th, 6th, 10th, and 24th postoperative weeks (*P* < 0.05) (Fig. 4).

### OGTT:

**Fig. 5:**
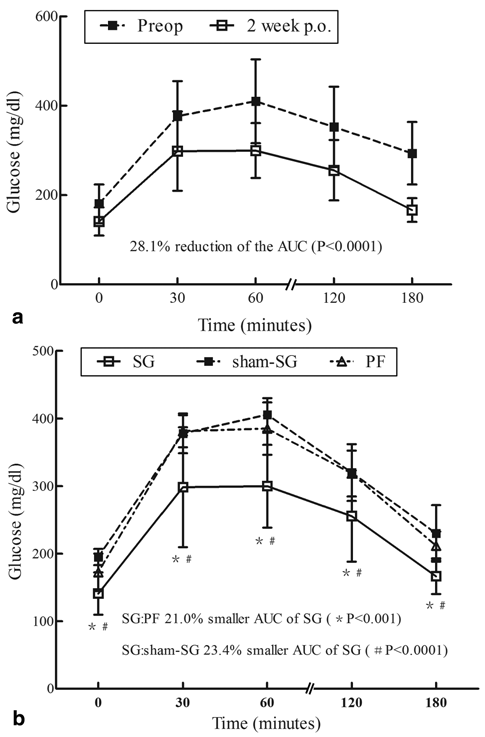
Glucose tolerance: **a** The oral glucose tolerance test performed in the sleeve gastrectomy (*SG*) rats in the two postoperative weeks indicated an improvement of glucose tolerance, 28.1 % reduction of *AUC* (*P* < 0.0001). **b** The *SG* group resulted in markedly better glucose tolerance comparing with that of the *sham-SG* and pair-fed (*PF*) groups. *SG* and *sham-SG* groups: 23.4 % smaller *AUC* in *SG* group (#*P* < 0.0001); *SG* and *PF* groups: 21.0 % smaller *AUC* in *SG* group (*P < 0.001)

Two weeks after surgical intervention, the SG group showed an improvement in glucose tolerance, and a significant reduction of the area under blood glucose concentration curve (AUC; by 28.1 %, *P* < 0.0001) was demonstrated in the Fig. 5a as well as a lower mean 30-min peak levels (298.4 ± 82.2 vs. 376.8 ± 51.6 mg/dl; *P* = 0.013) and a lower mean 120-min peak levels (255.7 ± 44.7 vs. 352.8 ± 76.8 mg/dl; *P* = 0.027) than the sham-SG groups. This significant effect could not be reproduced in PF and sham-SG groups, as the SG group had improved the glucose tolerance comparing with that in other groups (23.4 % smaller AUC of sham-SG, *P* < 0.0001; 21.0 % smaller AUC of PF, *P* < 0.001) (Fig. 5b).

### ITT:

**Fig. 6:**
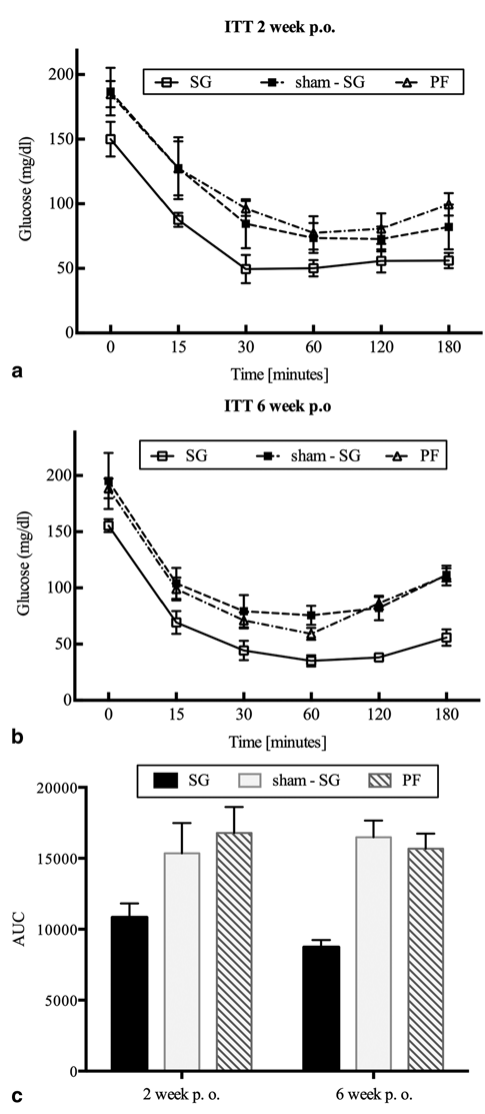
Both **a** and **b** show that the sleeve gastrectomy (*SG*) group improved insulin tolerance comparing with that of the *sham-SG* and pair-fed (*PF*) groups postoperative (*P* < 0.03), **c** shows the *SG* group significantly decreased the area under blood glucose concentration curve (*AUC*) comparing with that of the *sham-SG* and *PF* groups (*P* < 0.005)

The SG animals had lower levels of blood glucose (*P* < 0.03) (Fig. 6a)and smaller AUC (*P* < 0.005) than sham-SG and PF groups (Fig. 6b), which indicates better insulin sensitivity at the 2nd and 6th postoperative weeks.

### Hormones measurements ghrelin:

**Fig. 7:**
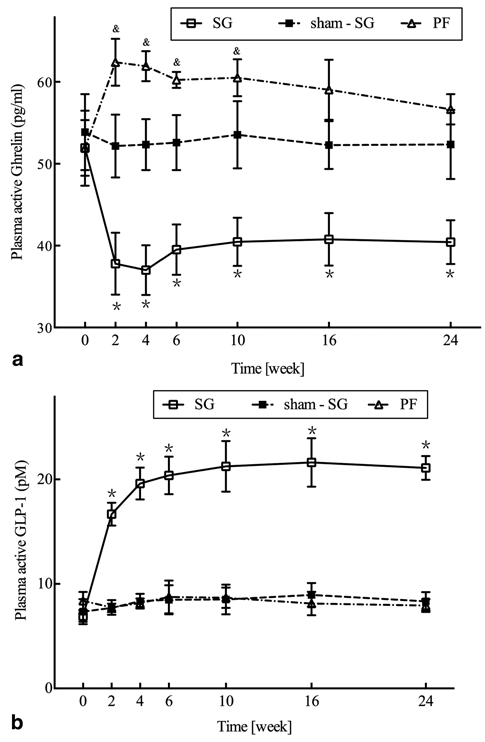
**a** Fasting plasma ghrelin: Mean fasting ghrelin level for the sleeve gastrectomy (*SG*) group was significantly lower than the *sham-SG* group (*P < 0.005). Before the 10th week, the fasting ghrelin level of the pair-fed (PF) group was increased compared with that of the *sham-SG* rats (& *P* < 0.05) throughout the whole period (24 weeks). **b** Plasma level of GLP-1 after oral glucose administration: The *SG* group was increased compared with that of the *sham-SG* and *PF* groups (*P < 0.0001)

In the process of the experiment, the ghrelin level was not significantly changed in the sham-SG group, but elevated in the PF group before the 10th week (*P* < 0.05). However, the ghrelin level subsequently decreased in the SG group during the examination period (*P* < 0.005, Fig. 7a).

### GLP-1:

It was shown that GLP-1 level for the sham-SG and the PF groups had no change throughout the entire period. The GLP-1 level of SG group had risen comparing with that of the sham–SG and the PF groups from the 2nd to 24th postoperative weeks (*P* < 0.0001) (Fig. 7b).

## Discussion

The SG was originally conceived as the first stage of achieving weight loss and reducing comorbidities in the patients who were superobese before undergoing a Roux-en-Y GBP or a BPD with duodenal switch (DS) [30]. However, SG was recognized as a stand-alone bariatric procedure particularly in the treatment of obese-related type 2 diabetes patients since approximately 8 years ago, as was demonstrated in a number of clinical and intervention studies [31]. The First International Consensus Summit for SG (2007) had proposed that SG is more than a gastric restrictive operation in improving or resolving T2DM and the MS [25] but with a similar effect as the GBP on inducing remission of T2DM patients [7]. However, former studies had a relatively short examination period and could not sufficiently explain the mechanisms behind the beneficial changes in glucose homeostasis. Our study, reveals that the SG group has a higher resolution rate of T2DM at 24 weeks after surgery in nonobese type 2 diabetic GK rats. It’s interesting to find that for the first 4 weeks after the surgery, although the SG and sham-SG groups had the similar weight loss, the blood glucose of the SG group decreased. The result above indicates that the glucose tolerance improved in the SG group compared with that in the sham-SG group. The weight loss in both the PF and SG groups had no difference throughout the experimental period, while the SG group had lower blood glucose levels compared with the PF group. Therefore, we verified the foregoing hypothesis as follows: In the early postoperative, SG can conduct direct effects on decreasing blood glucose, which is independent from weight loss and food intake [32, 33]. From the results above, we further proved that SG could play a direct hypoglycemic effect around the whole experimental period. The glucose tolerance in the SG group significantly improved compared with that in the PF groups, and this effect lasted until the 24th week in spite of the similar weight loss in the PF group. Among the SG, sham–SG, and PF groups, the fasting insulin concentration had no significant changes, which may be correlated with the change of GLP-1 concentration [34], whereas the HOMA-IR in the SG group had a remarkable reduction compared with that in the sham-SG and the PF groups during the whole experiment period. In our study, it can be suggested from the aforementioned data that an additive “gastric”, not weight and food intake loss-related mechanism contributes to the improvement of T2DM following SG operation. The rapid and remarkable improvement of insulin sensitivity observed in the SG group may be involved in the mechanism; however, the exact molecular mechanism of hypoglycemic is still unclear.

Many studies have examined the changes in the GI hormones, which may be the key factors of improving blood glucose [32, 33, 35]. Pories et al. [18], were the first to theorize the possibility of endocrine changes as a mechanism by which the GBP can effectively treat diabetes. A major gut hormone that has been identified as a member of incretin is the glucagon-like peptide-1 (GLP-1) that is produced by the small intestinal L-cells in response to fat and carbohydrates intake [36, 37]. The GLP-1 is capable of normalizing blood glucose, regulating insulin synthesis, and proinsulin gene expression as well as regulating the secretion of glucagon and somatostatin [38]. In our study, the GLP-1 level of SG group increased. Meanwhile, both the insulin sensitivity and fasting glucose level had improved. The above effects were not found in the PF group, which had no significant difference in the weight loss and food intake during the experimental period.

In addition to GLP-1, the change of ghrelin could also play an active role in beneficial glucose homeostasis after bariatric surgery. Ghrelin is an orexigenic hormone secreted primarily by the gastric fundus [39] that was shown to be a counter-regulatory hormone. Ghrelin can block the secretion of insulin [40] and block the release of the insulin-sensitizing peptide adiponectin [41]. Tong et al. [42], illustrated a robust proof-of-concept study that exogenous ghrelin administration reduced glucose-stimulated insulin secretion in healthy humans. Hence, Näslund et al. [43], indicated that ghrelin might have anti-incretin effects by counteracting GLP-1. More interestingly, Date et al. [44] found that ghrelin was also produced from islet α-cells and might affect b-cells through a paracrine action. In our study, compared with the sham-SG group, the ghrelin level of the SG group was significantly decreased, which may be mainly attributed to the cause of the resection of the gastric fundus [39]. The ghrelin level of PF group increased, which was possibly due to the effect triggered by the fasting state [45]. The SG and the PF groups had a similar weight loss or food intake throughout the experiment period; however, the SG group had better hypoglycemic effects. These results also suggest that SG might have direct improvement on the blood glucose level by decreasing the ghrelin level.

In summary, our study provided direct evidence that SG was not only a restrictive procedure, but also could have effects on blood glucose in the nonobese T2DM model rats independent from the effect of weight loss or food intake throughout the experimental period. The changes of the GLP-1 and ghrelin secretion may play an important role in controlling the T2DM. To extend our present findings and better understand the exact etiology of SG on the treatment of T2DM, further studies with larger sample, longer time follow-up are proposed, and the relationship between GI hormones are needed to be elaborated.
